# Attitudes and Needs of Health Care Providers Toward Artificial Intelligence–Assisted Pediatric Palliative Care: Mixed Methods Study

**DOI:** 10.2196/93400

**Published:** 2026-07-02

**Authors:** Siyu Cai, Qiaohong Guo, Zishen Wang, Ruixin Wang, Xuan Zhou, Xiaoxia Peng

**Affiliations:** 1 Center for Clinical Epidemiology and Evidence-based Medicine Beijing Children’s Hospital, Capital Medical University, National Center for Children's Health Beijing China; 2 School of Nursing Capital Medical University Beijing China; 3 Hematology Center, Beijing Children’s Hospital, Capital Medical University, National Center for Children’s Health Beijing China

**Keywords:** artificial intelligence, pediatric palliative care, attitudes, needs, mixed methods study

## Abstract

**Background:**

While artificial intelligence’s (AI’s) transformative potential in health care is widely acknowledged, its application in highly sensitive, humanistic domains like pediatric palliative care (PPC) remains largely unexplored.

**Objective:**

This study aims to explore the attitudes and needs of health care providers on the PPC assisted by AI, with the goal of informing future development and implementation of AI systems in this field.

**Methods:**

This was an explanatory sequential mixed methods study consisting of a nationwide cross-sectional questionnaire survey (March-April 2025) followed by qualitative semistructured interviews (August-October 2025). The quantitative study aimed to investigate PPC health care providers’ experiences, attitudes, and needs for the application of AI. Participants included team members of all recognized PPC teams in mainland China. The qualitative study aimed to explore in greater depth the potential future roles of AI in this field, as well as the features of an ideal AI-assisted tool for PPC. Potential interviewees were recruited from the pool of quantitative survey respondents.

**Results:**

Among 352 survey respondents, most (n=205, 58.24%) reported moderate familiarity with AI, with large language models being the most commonly used (n=280, 79.55%). Among large language model users, over half (161/280, 57.50%) reported using them for clinical purposes. Attitudes were generally positive: 67.05% (236/352) believed AI’s benefits would outweigh drawbacks, and 75% (264/352) considered its implementation feasible. The most desired applications were patient and family education (276/352, 78.41%) and symptom management (257/352, 73.01%). Interviews with 17 providers revealed three themes: (1) clinical roles and boundaries, (2) elements for clinical integration, and (3) challenges in development and deployment.

**Conclusions:**

This study reveals that PPC providers express positive attitudes and strong demand for AI-assisted clinical work. Furthermore, the research clarifies appropriate roles for AI, outlines elements for clinical integration, and highlights potential challenges in development and integration. This study provides evidence for the feasibility of AI application in PPC and offers guidance for the future development and deployment of AI tools.

## Introduction

Pediatric palliative care (PPC) is a family-centered, holistic care model dedicated to relieving distress, enhancing the quality of life, and providing comprehensive physical, psychological, social, and spiritual support to children with serious illnesses and their families throughout the disease trajectory [1]. Currently, this field faces numerous challenges, including resource limitations, difficulty in assessing and managing symptoms, the diverse and intricate nature of life-limiting pediatric conditions, ethically fraught decision-making, and the unique and evolving physiological, psychological, and social needs of children [2-5].

In recent years, the application of artificial intelligence (AI), particularly large language models, has experienced explosive growth in the medical field. AI has demonstrated considerable potential in areas such as medical image analysis, diagnostic support, electronic health record mining, and patient self-management [6]. The development of AI brings both exciting opportunities and unique challenges to PPC. AI has the potential to enhance PPC in several aspects, such as assessing patient needs, optimizing symptom management, assisting in medical decision-making, and even providing emotional support [7].

Despite the broad prospects for AI applications in the medical field, implementing AI technology in PPC faces unique challenges. Currently, the vast majority of medical AI research and applications focus on diagnosis and treatment, with their technological character and value orientation (such as pursuing efficiency and accuracy) fundamentally differing from the core principles of PPC [7,8]. Moreover, decision-making in PPC is highly contextualized. Health care providers need to consider multiple factors when making decisions, such as the child’s best interests, the family’s sociocultural background, and ethical considerations [9,10]. For example, clinical guidelines recommend that for children in the dying phase, nutritional support may be appropriately reduced or even withheld or withdrawn. Parents may be unable to accept, at either a cultural or an emotional level, the notion that their child could die in a state of hunger, perceiving the withdrawal of nutrition as an act of abandonment rather than an act of compassion. In such a scenario, the PPC team must simultaneously weigh the clinical evidence, the child’s current physiological state, the family’s cultural and spiritual values, and the profound emotional burden borne by the parents, ultimately arriving at an individualized nutritional strategy that honors both the child’s comfort and the family’s need for meaning and dignity in the dying process. This stands in stark contrast to the “standardized” decision-making based on evidence-based medicine that AI excels at handling.

The successful adoption and application of any technology ultimately depend on end-user acceptance and trust, alignment with clinical workflows, and the ability to effectively address practical pain points [11]. Existing studies mostly focus on the application of AI in general health care settings, focusing on treatment rather than improving quality of life. This knowledge gap hinders the design and implementation of AI systems that truly meet the needs of PPC. To address this gap, this study aims to systematically characterize PPC health care providers’ attitudes toward and needs for the application of AI in PPC. Using an explanatory sequential mixed methods design, we investigated the following: (1) PPC health care providers’ familiarity with, attitudes toward, and needs for AI applications in PPC; and (2) the potential future roles of AI in this field, as well as the features of an ideal AI-assisted tool for PPC. The findings are intended to provide evidence-based guidance for the future development and deployment of AI tools specifically tailored to the PPC context.

## Methods

### Study Design

This study used an explanatory sequential mixed methods approach, integrating quantitative data from a cross-sectional questionnaire with qualitative insights obtained through semistructured interviews. This study was initiated by the PPC subspecialty group of the Pediatrics Society of the Chinese Medical Association, the only professional organization for PPC in mainland China.

### Phase 1: Quantitative

Eligible participants were required to meet all the following criteria: (1) currently used as a health care provider (eg, physician, nurse, psychologist, social worker, or other allied health professional) delivering direct patient care within a recognized PPC team or unit in China; (2) at least one year of cumulative work experience in a health care setting; and (3) at least 6 months of experience providing PPC services. Participants were excluded from the study if they were in purely administrative or research roles without direct patient care responsibilities. The target population comprised all members of every recognized PPC team in mainland China.

Eligible PPC teams were first identified through a systematic screening process. The comprehensiveness of the sampling frame was ensured through three complementary identification strategies: the official member list of the PPC subspecialty group of the Pediatrics Society of the Chinese Medical Association, the list of PPC service teams compiled by nonprofit organizations, and snowball referrals from recognized PPC leaders. The convergence of these 3 independent sources minimized the likelihood of missing active teams. Relevant methodological information is detailed in a previously published study [4]. A total of 36 eligible PPC teams were included. The group leader of the PPC subspecialty sent the survey link to the team leaders of these eligible PPC teams. The team leaders, in turn, forwarded the link to all team members. The response rate was 68.62% (352/513).

The questionnaire included the following content: demographic characteristics; AI usage experience; attitudes toward the application of AI in PPC; and needs for AI applications in PPC. The detailed content of the questionnaire can be found in Multimedia Appendix 1. The development of the questionnaire was informed by previously published studies on attitudes and perceptions regarding the application of AI in various medical fields [12-14]. The initial item pool was reviewed by a panel of 3 PPC experts, who evaluated each item for relevance, clarity, and comprehensiveness; items were then revised based on their feedback. A pilot test involving 10 participants was conducted prior to general release; based on their feedback, item wording was clarified, and redundant response options were consolidated. The survey was conducted from March to April 2025, using the online questionnaire platform “Wenjuanxing.” Participants were requested to complete the survey within 2 weeks of receiving the invitation.

Data were analyzed using SAS (version 9.4; SAS Institute Inc.). Descriptive statistics were used to summarize the data. To examine whether attitudes and needs toward AI differed by professional role, subgroup analyses comparing physicians, nurses, and social workers were conducted using the Kruskal-Wallis test.

### Phase 2: Qualitative

Following the quantitative phase, a qualitative component was conducted to provide explanatory depth. Through semistructured interviews, this phase aimed to explore how future AI tools could be successfully integrated into PPC workflows. The focus was on identifying appropriate application areas and the essential conditions needed for AI to serve as a supportive, rather than intrusive, element in PPC practice.

The interview guide was developed by the research team based on the results of the quantitative study and informed by relevant literature. Interviews focused on three core topics: (1) Defining the role and boundaries of AI in PPC. (2) Attributes and deployment requirements for a PPC AI tool. (3) Challenges in development and deployment. The detailed interview outline can be found in Multimedia Appendix 2.

Potential interviewees were recruited from the pool of quantitative survey respondents. We selected individuals based on the following criteria: (1) Respondents who indicated they were “Very familiar” or “Somewhat familiar” with AI technology in the survey item “Are you familiar with AI technology?” (2) Respondents who reported using Large Language Models for “Assisting clinical work” in the survey item “Which tasks have you used Large Language Models for in your work?” A total of 31 survey respondents met both eligibility criteria and had voluntarily provided contact information (email or phone) at the end of the survey.

Purposive sampling was used to ensure maximum variation across key characteristics, including professional role, age group, and geographic region of practice. The principal researcher contacted eligible participants by sending a formal invitation detailing the study purpose, interview format, estimated duration, and the voluntary and confidential nature of participation. Of these, 5 did not respond. Interviews were conducted from August to October 2025 via Tencent Meeting. Each interview lasted 41-69 minutes (median 57). All interviews were audio-recorded with participant permission and transcribed verbatim.

Thematic analysis was conducted using Microsoft Word and Excel. The data coding and analysis proceeded through the following steps: (1) the primary researcher listened to all audio recordings and repeatedly read through the verbatim transcripts to achieve deep familiarity with the data prior to formal coding. (2) Two researchers (CS and ZX) independently generated initial codes from 3 interview transcripts, capturing the data relevant to the research questions. The two researchers (CS and ZX) subsequently met to compare their codes, discussing areas of agreement and divergence, and a preliminary coding framework was constructed. (3) Coded data were sorted and collated into potential themes. Codes were continuously compared, merged, split, or reorganized as additional transcripts were incorporated. (4) The research team reviewed themes to assess their internal coherence and their distinctiveness from one another, refining the thematic map accordingly. (5) The themes were further defined and named through ongoing analytical discussion among the research team. Regular team meetings were held to discuss, validate, and revise themes and codes until consensus was reached. (6) A theme table was constructed in Excel to present themes, subthemes, and supporting quotations. Participants’ original words were retained wherever possible for key expressions, followed by descriptive analysis. Recruitment and analysis proceeded concurrently. Saturation was defined as the point at which no new codes, themes, or conceptual dimensions emerged from additional interviews; recruitment was concluded when 3 consecutive interviews yielded no new codes, themes, or conceptual dimensions, which occurred at interview 17.

### Mixed Methods Integration

In this explanatory sequential mixed methods study, integration occurred at 2 primary points. First, the quantitative findings from Phase 1 directly informed the research focus and design of phase 2. The phase 1 results demonstrated that PPC health care providers held broadly positive attitudes toward AI and expressed demand for its application across a wide range of clinical domains. This conclusion generated a natural next question: given that AI application in this field is both wanted and considered viable, what should an ideal AI tool for PPC actually look like in practice? This question could not be adequately addressed through a structured questionnaire alone, as it required in-depth exploration of nuanced clinical contexts, professional values, and implementation realities. Accordingly, phase 2 used a semistructured interview to explore 3 dimensions of an ideal AI tool: its appropriate clinical roles and boundaries, the attributes and deployment conditions required for successful integration, and the challenges that must be anticipated in development and implementation. Interviewees were purposively selected from phase 1 survey respondents with direct AI experience in clinical settings. Second, quantitative and qualitative data were interpreted in conjunction to complement and expand upon each other, generating integrated conclusions. Qualitative findings served to explain, expand, and contextualize the quantitative results.

### Ethical Considerations

This study was approved by the Institutional Review Board at Capital Medical University (number Z2025SY024). For the quantitative study, participants were notified that returning the survey implied informed consent. No names or other identifying information were collected from participants, ensuring the responses were anonymous. All data were held securely and confidentially, and only accessed by the research team. For the qualitative study, all of the participants provided informed consent.

## Results

### Phase 1: Quantitative

#### Demographic Characteristics

A total of 352 participants were recruited from PPC teams across mainland China. The demographic characteristics of the participants are summarized in Table 1. The median age was 37 (IQR 32-42) years. The median total work experience was 12 (IQR 7.25-18) years, while the median duration of specific experience in PPC was 3 (IQR 1-5) years.

**Table 1:** Demographic characteristics of participants.

Characteristics				Participants, n (%)
Department				
		Hematology and oncology department	177 (50.28)	
		Pain management department	13 (3.69)	
		Pediatrics department	81 (23.01)	
		Critical care medicine	15 (4.26)	
		Palliative care or hospice department	0 (0)	
		Other	66 (18.75)	
Sex				
	Male		64 (18.18)	
	Female		288 (81.82)	
Title				
		Senior title	31 (8.81)	
		Associate senior title	68 (19.32)	
		Intermediate title	155 (44.03)	
		Junior title	77 (21.88)	
		None	21 (5.97)	
Academic degree				
		Doctor	25 (7.1)	
		Master	140 (39.77)	
		Bachelor	165 (46.88)	
		Other	22 (6.25)	
Profession				
		Physician	134 (38.07)	
		Nurse	139 (39.49)	
		Social worker	38 (10.8)	
		Psychologist	11 (3.13)	
		Other	30 (8.52)	

#### AI Usage Experience

Participants’ usage experience of AI is shown in Table 2. The survey results revealed varying levels of familiarity with AI among participants. Fifty-one (51/352, 14.49%) reported being very or somewhat familiar with AI, while the majority (205/352, 58.24%) indicated a moderate level of familiarity. Large language models (280/352, 79.55%) and intelligent voice assistants (279/352, 79.26%) were the most widely adopted technologies. In contrast, health care-specific AI tools such as clinical decision support systems (95/352, 26.99%), medical virtual assistants (76/352, 21.59%), and medical image analysis tools (52/352, 14.77%) were less commonly used. Among large language model users (n=280), over half (161/280, 57.50%) reported using them for clinical purposes.

**Table 2:** Usage experience with artificial intelligence.

Questions			Participants, n (%)
Are you familiar with AI^a^ technology?			
	Very familiar	3 (0.85)	
	Somewhat familiar	48 (13.64)	
	Moderately familiar	205 (58.24)	
	Slightly familiar	84 (23.86)	
	Not familiar at all	12 (3.41)	
Which AI technologies have you used in your work or daily life?			
	Large language models	280 (79.55)	
	Intelligent voice assistants	279 (79.26)	
	Clinical decision support systems	95 (26.99)	
	Medical virtual assistants	76 (21.59)	
	Medical image analysis tools	52 (14.77)	
	Other	0 (0)	
	None of the above	11 (3.13)	
Which tasks have you used large language models for in your work or daily life? (n=280)			
	Solving daily life problems	224 (80)	
	Assisting clinical work	161 (57.50)	
	Assisting with scientific research work	129 (46.07)	
	Other	13 (4.64)	

^a^AI: artificial intelligence.

#### Attitudes Toward the Application of AI in PPC

The participants’ attitudes toward AI application in PPC are detailed in Table 3. Participants generally demonstrated positive attitudes toward the application of AI in PPC. A substantial majority (236/352, 67.05%) believed that the positive impacts of AI would outweigh its negative effects, and 41.76% (147/352) expressed strong support for its integration into PPC services. Regarding feasibility, 75% (264/352) of participants considered the implementation of AI in PPC to be highly or somewhat feasible.

In terms of perceived advantages, improved work efficiency was identified by the largest proportion of respondents (284/352, 80.68%). However, several barriers were noted, with insufficient technological accuracy being the most frequently cited concern (240/352, 68.18%). Similarly, the primary concern regarding AI implementation was the potential for AI to provide incorrect information (239/352, 67.90%). Only a minority (86/352, 24.43%) believed that AI may replace PPC staff in the future.

**Table 3:** Attitudes toward the application of artificial intelligence in pediatric palliative care.

Questions			Participants, n (%)
What impact do you think AI^a^ will have on the field of PPC^b^?			
	Positive impact outweighs negative impact	236 (67.05)	
	Negative impact outweighs positive impact	9 (2.56)	
	Uncertain	107 (30.4)	
Do you support the use of AI to assist in PPC services?			
	Strongly support	147 (41.76)	
	Somewhat support	131 (37.22)	
	Neutral	64 (18.18)	
	Somewhat oppose	7 (1.99)	
	Strongly oppose	3 (0.85)	
How feasible do you think the application of AI in PPC is?			
	Highly feasible	106 (30.11)	
	Somewhat feasible	158 (44.89)	
	Moderately feasible	82 (23.3)	
	Somewhat infeasible	6 (1.70)	
	Completely infeasible	0 (0)	
What do you think are the advantages of applying AI in PPC?			
	Improves work efficiency	284 (80.68)	
	Helps promote personalized care	261 (74.15)	
	Lightens the workload of health care workers	235 (66.76)	
	Reduces labor costs	225 (63.92)	
	Reduces health care costs	205 (58.24)	
	Improves patient and family satisfaction	186 (52.84)	
	Reduces medical errors	144 (40.91)	
	Promotes resource allocation	142 (40.34)	
	Enhances communication with patients and families	134 (38.07)	
	Other	0 (0)	
What do you think are the barriers to applying AI in PPC at the current stage?			
	Insufficient accuracy of technology	240 (68.18)	
	Technology cannot handle complex clinical situations	236 (67.05)	
	Ethical and legal issues	213 (60.51)	
	Lack of policies, funding, and technical resources required for implementing AI technology	190 (53.98)	
	Health care workers lack professional knowledge and training in AI	182 (51.70)	
	Lack of transparency or explainability in technology	169 (48.01)	
	Technology fails to ensure fairness	145 (41.19)	
	Parents' and patients’ acceptance is low	115 (32.67)	
	Health care workers’ acceptance is low	56 (15.91)	
	Other	3 (0.85)	
What aspects of applying AI technology in PPC services concern you?			
	AI may provide incorrect information	239 (67.90)	
	Difficulty explaining AI algorithms and decision-making processes (“black box” problem)	234 (66.48)	
	Lack of human compassion in medical services	229 (65.06)	
	Difficulty ensuring patient information and privacy security	222 (63.07)	
	Relying on AI may lead to skill degradation among health care workers	127 (36.08)	
	AI may fail to ensure fairness	118 (33.52)	
	AI may exacerbate regional disparities in health care resources and service	112 (31.82)	
	AI may replace PPC staff in the future	86 (24.43)	
	Other	0 (0)	

^a^AI: artificial intelligence.

^b^PPC: pediatric palliative care.

#### Needs for AI Applications in PPC

Participants expressed a strong demand for AI applications in PPC (Table 4). The most desired areas for AI implementation were patient and family education (276/352, 78.41%) and symptom management (257/352, 73.01%). Notably, an overwhelming majority (322/352, 91.48%) considered a clinical decision support system for PPC to be very or somewhat necessary.

**Table 4:** Need for artificial intelligence applications in pediatric palliative care.

Questions			Participants, n (%)
In PPC^a^, which services would you like AI^b^ to improve?			
	Patient and family education	276 (78.41)	
	Symptom management	257 (73.01)	
	Psychological and spiritual support for family members	246 (69.89)	
	Palliative care referrals	229 (65.06)	
	Assessment and identification of patient needs	226 (64.2)	
	Psychological and spiritual support for the patient	206 (58.52)	
	Social support	173 (49.15)	
	Doctor-patient communication	165 (46.88)	
	Home care	162 (46.02)	
	Bereavement support	159 (45.17)	
	Other	0 (0)	
Do you need a clinical decision support system for PPC to assist you in making decisions?			
	Very necessary	214 (60.80)	
	Somewhat necessary	108 (30.68)	
	Moderately necessary	24 (6.82)	
	Slightly necessary	4 (1.14)	
	Not necessary	2 (0.57)	

^a^PPC: pediatric palliative care.

^b^AI: artificial intelligence.

#### Subgroup Analyses

We analyzed differences in AI familiarity and attitudes across professional groups (physicians, nurses, and social workers). Statistically significant differences were observed in the level of familiarity with AI technology (χ²_2_=12.47; *P*=.002), with social workers reporting the highest familiarity. Significant differences were also found in the degree of support for AI-assisted PPC services (χ²_2_=16.56; *P*<.001), with nurses demonstrating the highest level of support. Similarly, assessments of the feasibility of AI application in PPC differed significantly across professional groups (χ²_2_=15.95; *P*<.001), with nurses rating feasibility most favorably.

### Phase 2: Qualitative

**Figure 1:**
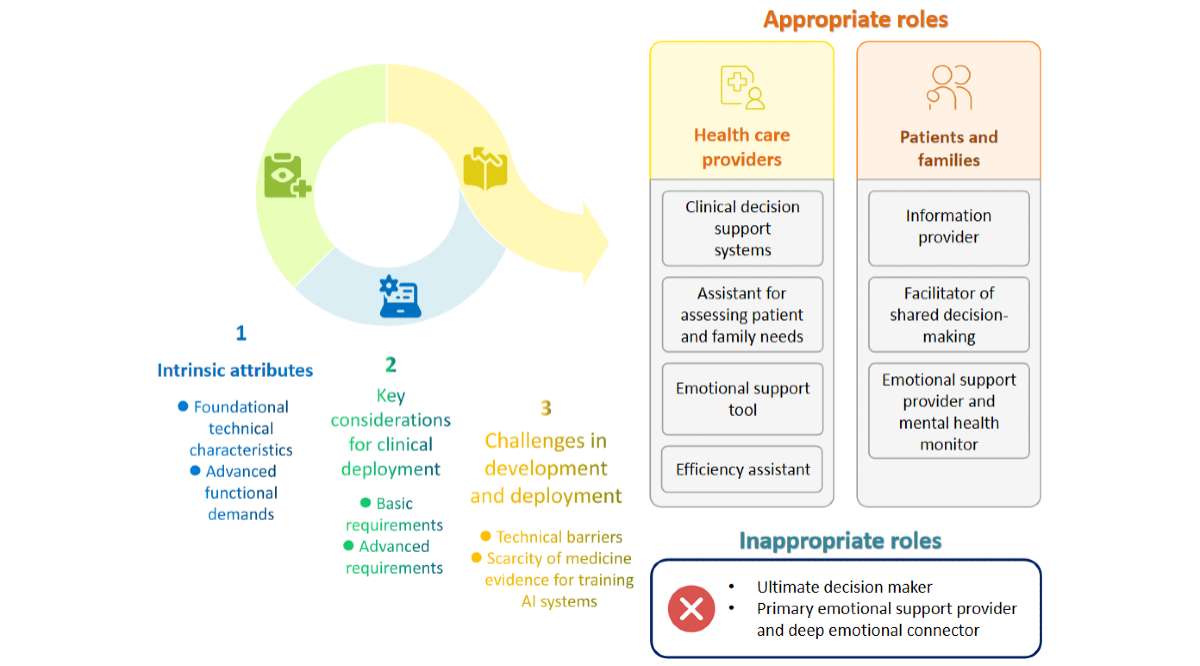
Key points for integrating an artificial intelligence (AI) tool in pediatric palliative care.

Figure 1 illustrates the key points for integrating AI tools in PPC, including their defined roles, necessary implementation elements, and challenges. The quotations for main themes and subthemes are presented in Table 5.

**Table 5:** Quotations for main themes and subthemes.

Main themes and subthemes			Quotations
Clinical roles and boundaries			
	Appropriate roles and their functions	Physician 1: I need it (AI^a^) to give me some guidance, for example, on the usage and dosage of medications. Social worker 1: AI can collect information and do assessments. It can integrate sensing technologies, like smart mattresses, wearable devices, and so on. For instance, which part of the body is in contact with the bed, for how long, and with how much pressure. This information can be used to prevent bedsores. Physician 3: I think many parents would need to know, if their child has certain symptoms and want him/her to be hospitalized, which hospitals can admit him/her, and what exactly are the contact details. That’s what they need... What patients need more is information, like information about the disease, about how to treat it, about how to take the medication, about how to cook for patient. Nurse 3: As long as the patient has a phone, they can look up a lot of information. We once had a nine-year-old with melanoma. He searched online and found out the mortality rate was pretty high. When I was taking him for tests, while his parents weren’t around, he held my hand and asked me, ‘Am I going to die soon?’	
	Inappropriate roles and their functions	Physician 3: It (AI) cannot lead the decision-making process. The one who should ultimately be responsible for decisions is the doctor. Physician 5: AI can give you a diagnosis, but it can never replace me actually being there beside you, giving you a real, warm hug.	
Elements for clinical integration			
	Intrinsic attributes of the tool	Physician 1: A child can’t eat and keeps throwing up, but the parents just can’t accept that their kid isn’t eating anything. What should we do? We need AI to give me some ways to think through this, ideally by offering me multiple options.	
	Key considerations for clinical deployment	Physician 2: Training is really important. The quality of the input directly shapes the quality of the output.	
Challenges in development and deployment			
	Technical barriers	Social worker 2: I don’t think current AI tech is really there yet, you know, when it comes to actually understanding and pulling together all kinds of data like clinical indicators, what patients say, doctor-patient communication, and stuff like that.	
	Scarcity of evidence for training AI systems	Physician 4: A lot of times, what we know about using medicines is really just a feeling we get from working with patients. It’s hard to turn that into the kind of proof that evidence-based medicine needs. For instance, in my own experience, with a drug like midazolam, I’ve found that for patients with heart problems, even a very small dose can make them become sedated very quickly.	

^a^AI: artificial intelligence.

#### Demographic Characteristics

A total of 17 health care providers participated in the interviews, comprising 7 physicians, 6 nurses, and 4 social workers. Their average age was 43.18 (SD 9.17) years, with an average of 7.06 (SD 3.17) years of experience in palliative care.

### Theme 1: Clinical Roles and Boundaries

#### Appropriate Roles and Their Functions

Participants perceived AI as capable of serving as a high-quality clinical decision support system. For structured problems (eg, medication dosing), AI could offer information and guidance based on the latest evidence and guidelines. For unstructured, complex decision-making problems with no standardized answers (eg, prognosis discussions), AI could offer thinking frameworks, past case examples, and suggestions on practice to inform health care providers’ decision-making processes.

Participants regarded AI as an assistant that improves efficiency by handling tasks such as medical documentation, organizing family communication records, and other administrative tasks.

Participants also indicated that AI could serve as an assistant for assessing the needs of pediatric patients and their families, with particular potential in evaluating children’s needs. Since children (especially younger children or those who are critically ill) often cannot accurately describe their own feelings and needs, AI could collect and analyze multimodal data to provide objective assessment references for assessing experiences such as pain and anxiety. AI was perceived as enabling continuous symptom monitoring, particularly in home-based care environments, with the ability to provide timely alerts for medical intervention when necessary. Furthermore, in terms of communication, AI could assist health care providers in interpreting the underlying needs and emotional states conveyed through children’s limited verbal expressions.

Health care providers in PPC bear a significant burden of emotional labor. Participants noted that AI could function as an emotional support tool, offering timely, continuous, and professional psychological guidance to help prevent mental health issues and professional burnout.

Regarding patients’ families, participants believed AI could take on the role of an information provider. Tailored to the child’s specific diagnosis, developmental stage, phase of treatment, and family preferences, AI could deliver personalized information support, covering all dimensions of care, including disease explanation, caregiving skills, social resources, and so on.

Parents could obtain more disease-related information through AI, which helped them participate in shared decision-making. However, parents might also cross-check the information provided by health care providers against AI-generated content. If a health care provider could not adequately address the questions raised by parents, it had the potential to strain the doctor-patient relationship. For children, a significant impact of AI technology was that it allowed pediatric patients to access information about their own condition directly, bypassing adults (such as parents and doctors). On one hand, this could enhance children’s right to information and autonomy. On the other hand, unscreened and unprocessed medical information might exceed a child’s capacity to understand and handle it. Health care providers needed to learn how to help children interpret and cope with the information they accessed.

For family members, AI could provide round-the-clock emotional support and companionship to alleviate feelings of isolation and anxiety. Simultaneously, AI systems could continuously monitor and assess the mental health status of both patients and family members, analyzing patterns in conversations to detect early signs of emotional distress. When concerning patterns were identified, the AI could alert health care providers or recommend appropriate mental health resources, ensuring timely interventions and comprehensive care for the entire family unit.

#### Inappropriate Roles and Their Functions

Participants emphasized that AI must never assume ultimate responsibility for clinical decision-making. The final accountability for clinical decisions must rest with qualified health care providers.

Participants believed that in the dimensions of emotional support and relationship-building within PPC, human health care providers held an irreplaceable position. PPC was not merely about delivering medical services; it was also about establishing deep emotional connections with the child and their family to support them in enduring the challenges brought by the illness. The formation of such relationships depended on human authenticity and shared life experiences. While AI could analyze, mimic, and even respond to emotions, it could not provide the comfort that only another living, empathetic being of flesh and blood can offer.

### Theme 2: Elements for Clinical Integration

#### Intrinsic Attributes of the Tool

Participants universally emphasized that an AI tool must possess a set of foundational technical characteristics, including accuracy, transparency, explainability, real-time capability, safety, privacy protection, ethical compliance, and so on.

Beyond these basic requirements, participants articulated more specialized and in-depth functional demands, based on the highly individualized nature of PPC and its need to handle complex situations. The foremost demand raised was for personalized decision support. Participants indicated that AI needed to integrate multidimensional data, such as cultural background, disease status, and personal preferences, to generate tailored decision recommendations. In addition, when dealing with unstructured, complex decisions (eg, end-of-life communication and ethical dilemmas), participants expressed a desire for AI to offer thinking frameworks rather than definitive solutions. For decisions with no single correct answer, they suggested that AI should present multiple possible approaches along with an analysis of their potential outcomes.

#### Key Considerations for Clinical Deployment

Participants identified the following elements as critical for the successful integration of an AI tool into clinical workflows: seamless information system integration, ease of use, provision of adequate training, features that facilitate intra-team collaboration, and support in the financial, policy, and legal domains. Additionally, participants emphasized that AI should support multiple input and output methods such as voice, text, and image to adapt to different scenario requirements.

### Theme 3: Challenges in Development and Deployment

#### Technical Barriers

AI tools require the integration of heterogeneous data and the aggregation of information from diverse origins, rendering their development technically demanding. Furthermore, children, especially younger or critically ill patients, face inherent limitations in expressing their feelings and needs. They could only convey information indirectly through facial expressions, crying, body movements, or simple words. This posed higher-level technical challenges for the development of AI tools.

#### Scarcity of Evidence for Training AI Systems

Participants highlighted a scarcity of high-quality medical evidence suitable for training AI systems. This shortage was attributed to the unique characteristics and vulnerability of the PPC patient population. In addition, participants stated that many decisions in this field relied on physicians’ experience accumulated through long-term practice, including intuition regarding subtle symptom changes and a keen understanding of family dynamics. Such deeply contextual, experience-based knowledge was difficult to distill into standardized, evidence-based guidelines.

## Discussion

### Principal Findings

This study revealed an overall positive attitude and clear demand for AI technology among health care providers in the PPC field, laying an important foundation for its application. This positive stance may stem from several factors: (1) the practical need to improve work efficiency; (2) the backdrop of strained human resources and excessive workloads in PPC; (3) AI’s potential to enhance decision-making quality and even assist in addressing clinical dilemmas; and (4) AI’s ability, from the perspective of family care, to compensate for current service gaps by offering more comprehensive, continuous, and personalized support to families [15-17]. Furthermore, the finding that only 24.43% (86/352) of participants believe AI will replace their roles may contribute to this high level of acceptance, suggesting that most see AI as a complementary tool rather than a threat to their profession.

The broadly positive attitudes toward AI integration identified in this study are consistent with findings from comparable international research. Lambert et al.’s integrative review of 42 studies across multiple countries documented similarly favorable attitudes among health care providers, particularly when AI tools were perceived as augmenting rather than replacing human judgment [18]. Across diverse national contexts, health care providers’ acceptance of AI has been consistently associated with perceived usefulness and ease of use that appear to transcend national boundaries and suggest a degree of convergence in professional attitudes toward AI-assisted care [19].

Despite the overall positive attitudes observed, the integration of AI into PPC still faces substantial risks. First, algorithmic bias may produce recommendations that are inequitable or clinically inappropriate [20]. Second, over-reliance on AI tools may lead to deskilling, wherein health care providers defer inappropriately to algorithmic outputs even when these conflict with clinical judgment [21]. Therefore, ongoing auditing of AI outputs against real-world clinical outcomes is necessary. Third, confidentiality concerns are heightened in PPC, necessitating robust data encryption, strict access controls, and informed consent processes regarding AI-mediated data handling [22]. Taken together, these considerations suggest that the path toward AI-assisted PPC is not solely a technical challenge but equally a governance and cultural one, requiring sustained collaboration among health care providers, technology developers, ethicists, patients, and families to ensure that AI serves as an instrument of humane care rather than a source of new inequities or harms [23].

Given the breadth and diversity of AI’s potential applications in PPC, it is particularly important and urgent to clearly define its scope of use and establish appropriate boundaries [24]. This study clearly indicates that health care providers welcome AI as an efficient auxiliary tool. However, they also firmly advocate that its role should be strictly limited to that of an “assistant” and “information provider,” without encroaching upon the core domains of human professional judgment and compassionate care. This cautious attitude toward technological application is not rooted in resistance to innovation, but in a deep understanding of PPC services’ essence. It reflects the belief that any introduced technology must enhance, rather than undermine, the fundamental principle of family-centered, holistic care [25,26].

The application of AI in palliative care differs significantly from its use in other medical fields. Unlike domains where AI primarily focuses on diagnosis and treatment, the goal of AI in palliative care is to enhance quality of life. This shift in objective not only expands the boundaries of AI’s application in health care but also presents new challenges to its technological architecture and implementation paradigms [25,27]. Specifically, AI tools in the PPC field must be capable of collecting and synthesizing heterogeneous data streams, such as clinical records, natural language inputs from family communications, and behavioral or physiological signals from pediatric patients. Moreover, rather than delivering uniform, standardized clinical recommendations, participants in this study emphasized the need for AI to provide personalized decision support, offering structured thinking frameworks and multiple alternative pathways for complex decisions that lack definitive answers. In addition, given that younger or critically ill children are frequently unable to verbally communicate their experiences, AI systems must also be capable of recognizing and interpreting both verbal and nonverbal signals, including vocalizations, facial expressions, body movements, and physiological responses. Taken together, these demands translate into concrete technical requirements for AI development in PPC: multimodal fusion architectures capable of integrating structured and unstructured data from diverse sources; adaptive, context-sensitive reasoning mechanisms that generate individualized decision support; and multimodal perception modules incorporating natural language processing, computer vision, and sensor-based inference to decode the complex communicative signals of pediatric patients [28,29].

Training for health care providers is one of the essential strategies for AI deployment. This necessity stems from multiple factors. Primarily, health care providers must familiarize themselves with these innovative tools to seamlessly integrate them into their daily practices. Additionally, they need to develop the critical skills to identify and mitigate potential errors or biases that may emerge from AI-generated outputs [30,31]. More critically, AI is transforming the physician-patient relationship. In this new landscape, health care providers must navigate situations where patients have unprecedented access to medical information via AI [32]. This shift requires health care providers to develop new skills in interpreting AI-generated insights and communicating them effectively to patients and families, all while maintaining the empathetic, personalized care that is central to PPC [33,34].

### Limitations

This study was limited to PPC team members within China; variations in cultural norms, team composition, and health care system organization across different national contexts may influence providers’ attitudes toward and needs for AI, potentially limiting the generalizability of our findings to other settings. Additionally, this study used a cross-sectional survey design, which captures attitudes at a single point in time; as AI technology continues to evolve and health care providers’ familiarity with these tools increases, attitudes and perceived needs may shift accordingly. Furthermore, participants for the qualitative phase were purposively selected on the basis of their familiarity with AI, given that those without prior AI experience would have been unable to meaningfully engage with the interview questions. While this approach ensured the feasibility of data collection, it may therefore not fully reflect the views of PPC professionals with limited AI experience.

### Conclusions

To our knowledge, this is one of the first studies to systematically explore the specific needs and expectations of PPC health care providers regarding AI. This study reveals that health care providers in PPC hold a positive attitude toward AI, expecting it to play a supportive role across multiple aspects of care. It provides important evidence-based support for the feasibility of AI deployment in this field. Furthermore, the research clarifies appropriate roles for AI, outlines elements for clinical integration, and highlights potential challenges in development and integration. These insights provide clear evidence and guidance for the technological development and clinical adoption of AI in PPC.

## Appendix

Multimedia Appendix 1 Questionnaire.

Multimedia Appendix 2 Interview guide.

## Abbreviations

AI: artificial intelligence
PPC: pediatric palliative care

## Data Availability

The data that support the findings of this study are available on request from the corresponding author. The data are not publicly available due to privacy or ethical restrictions.
