# Supplementary material for: Attitudes and Needs of Health Care Providers Toward Artificial Intelligence–Assisted Pediatric Palliative Care: Mixed Methods Study
Source: J Med Internet Res. 2026 Jul 2;28:e93400. doi: 10.2196/93400 (PMC13376846; doi:10.2196/93400)
Supplement: Multimedia Appendix 1 [file jmir_v28i1e93400_app1.docx]

Appendix 2. Interview Guide

1. In your current practice, what specific clinical problems have you used AI to assist with, and how would you describe your overall experience?

2. Looking ahead, where do you see the potential for AI to assist in the future daily work of PPC? In clinical practice, what role would you ideally want AI to play? What specific challenges would you hope it could help you address? What problems do you envision it helping patients and their families solve?

3. In what scenarios within PPC do you believe AI would be unsuitable or inappropriate?

4. What features do you value most in potential AI tools? How would you like AI tools to integrate with existing workflows and systems? Do you have any preferences or suggestions regarding data input and output?

5. What major obstacles or challenges do you foresee in implementing AI tools in the clinical setting?

6. Are there any other perspectives, concerns, or insights regarding AI in healthcare that you would like to share?
